# From mechanisms to therapies: exploiting epigenetic and post-translational modifications of mitochondrial quality control in diabetic kidney disease

**DOI:** 10.3389/fendo.2026.1821507

**Published:** 2026-05-08

**Authors:** Dan-mai Zhao, Zhong-hong Yan, Yi-yang Yu, Yang-yang Zhang, Zhong-qiu Luan

**Affiliations:** 1 First Affiliated Hospital of Heilongjiang University of Chinese Medicine, Harbin, Heilongjiang, China; 2 Heilongjiang University of Traditional Chinese Medicine, Harbin, Heilongjiang, China

**Keywords:** diabetic kidney disease, DNA methylation, epigenetics and post-translational modifications, mitochondrial quality control, mitophagy, ubiquitination

## Abstract

Diabetic kidney disease (DKD) is a major and severe microvascular complication of diabetes and one of the primary causes of end-stage renal failure. As the body’s largest metabolic organ, the kidneys require a continuous supply of energy to maintain systemic homeostasis and normal metabolic functions. The mitochondrial quality control (MQC) system plays a central role in preserving cellular energy homeostasis by regulating key processes such as mitochondrial biogenesis, dynamics, and mitophagy, which is particularly critical for the highly energy-demanding kidneys. Emerging evidence indicates that epigenetic regulation—including DNA methylation, histone modifications, and non-coding RNA interactions—along with diverse post-translational modifications (PTMs) such as phosphorylation, ubiquitination, methylation, and acetylation, are deeply involved in the fine-tuning of MQC. These regulatory mechanisms significantly contribute to the pathogenesis and progression of DKD. This review systematically summarizes the interplay between MQC, epigenetic regulation, and PTMs, with a focus on how they collectively influence the course and outcome of DKD. Furthermore, it outlines recent advances in therapeutic strategies targeting this regulatory network, aiming to provide novel insights and future research directions for targeted interventions in DKD.

## 1 Introduction

Diabetic kidney disease (DKD), a prevalent microvascular complication of diabetes, affects approximately 22–40% of diabetic patients. It represents a leading cause of chronic kidney disease (CKD) and is a major contributor to end-stage renal disease (ESRD) (1). The kidneys are vital organs that play a central role in maintaining physiological homeostasis. Their primary functions involve the elimination of metabolic waste products through glomerular filtration, followed by the reabsorption of water, ions, and nutrients from the glomerular filtrate in the proximal tubules and the thick ascending limbs of Henle’s loop. These processes are essential for preserving the body’s internal environment and supporting normal metabolic activity (2). DKD is driven by hyperglycemia, hypertension, and epigenetic factors. Its natural progression typically involves initial glomerular hyperfiltration, followed by the development and advancement of albuminuria, a subsequent decline in glomerular filtration rate (GFR), and eventual progression to ESRD (3). The reabsorption of solutes by renal tubular cells is an energy-intensive process, requiring a substantial amount of ATP to sustain normal function. As a result, the kidneys are second only to the heart in terms of oxygen consumption and mitochondrial content in the human body (4, 5). Mitochondria are abundant in proximal and distal tubules located in the renal cortex, whereas tubular cells of the collecting duct and loop of Henle in the medulla contain relatively few mitochondria (6). Podocytes exhibit an intermediate mitochondrial density and a metabolically flexible phenotype, utilizing both oxidative phosphorylation and glycolysis to meet their energy demands (7, 8). However, the pathogenesis of DKD is complex, and existing prevention and treatment methods have limited efficacy in reducing its incidence and mortality.

Mitochondria are double-membrane organelles that function as the cell’s powerhouses. They generate adenosine triphosphate (ATP) through oxidative phosphorylation (OXPHOS), which accounts for over 90% of the body’s energy production (9). In addition to ATP synthesis, the electron transport chain (ETC) is the primary source of reactive oxygen species (ROS). While moderate levels of ROS act as signaling molecules, excessive ROS production, when it surpasses the capacity of antioxidant defense systems, leads to oxidative stress (OS) (10). Beyond their role in energy metabolism, mitochondria are also critical regulators of apoptosis, initiating intrinsic cell death pathways through the release of pro-apoptotic factors such as cytochrome c (11). To cope with increased metabolic demands and stress, cells depend on the mitochondrial quality control (MQC) system to preserve mitochondrial integrity. The MQC system represents a multilayered regulatory network that plays a pivotal role in maintaining mitochondrial functionality and homeostasis. It orchestrates a series of interconnected processes, including mitochondrial biogenesis, fission, fusion, protein degradation, and mitophagy, to ensure a healthy mitochondrial population within the cell. Disruption of any component of the MQC network can result in mitochondrial dysfunction, ultimately compromising cellular and tissue health (12, 13). Therefore, maintaining mitochondrial homeostasis and quality control is essential for preserving normal kidney function.

Increasing evidence suggests that MQC is tightly regulated at multiple levels, including through post-translational modifications (PTMs) and epigenetic modifications (14). Epigenetic modifications represent a dynamic and reversible process, encompassing DNA methylation, chromatin remodeling, histone modifications, and the regulation of non-coding RNAs (ncRNA). PTMs include phosphorylation, ubiquitination, SUMOylation, acetylation, and other modifications (15, 16). These mechanisms and modifications, whether acting independently or synergistically, influence chromatin structure and accessibility, thereby responding to various signals that regulate gene expression (17). Epigenetics is a rapidly evolving field, with numerous studies in recent years demonstrating that epigenetic modifications and PTMs play significant roles in the development of various kidney diseases (18). Research indicates that multiple microRNAs, long non-coding RNAs (lncRNAs), DNA methylation, and histone modifications are significantly associated with key clinical parameters such as urinary albumin excretion rate, eGFR, glycated hemoglobin A1c (HbA1c), and creatinine levels in patients with DKD (19–21). Furthermore, epigenetics and PTMs can also impact DKD by modulating MQC.

This review first summarizes the regulatory mechanisms of MQC in DKD, with a particular focus on mitochondrial biogenesis, dynamics, and mitophagy. It then provides a concise overview of epigenetic processes, including DNA methylation, PTMs, and ncRNA regulation. Subsequently, the review details the impact of epigenetic regulation and post-translational modifications in MQC on DKD. Finally, we summarize the latest approaches for targeted regulation of MQC through epigenetic drugs in the treatment of DKD, followed by a discussion of current research limitations and the proposal of promising therapeutic strategies, aimed at uncover additional underlying mechanisms of DKD and provide insights for future research and prevention strategies.

## 2 Mitochondrial quality control in DKD

**Figure 1:**
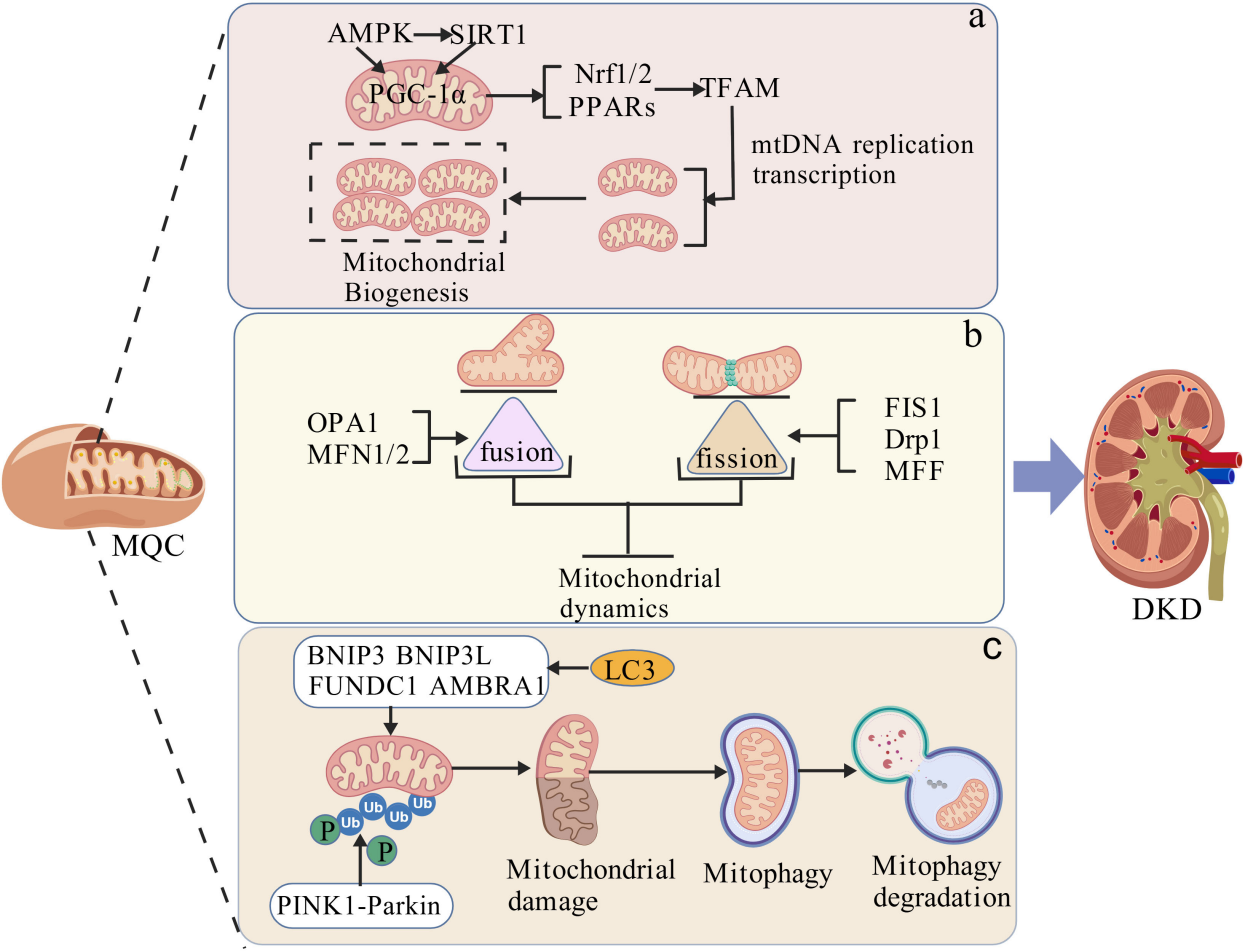
The mechanistic impact of mitochondrial quality control on diabetic kidney disease. **(a)** Effects of mitochondrial biogenesis on DKD; **(b)** Effects of mitochondrial dynamics regulation on DKD; **(c)** Effects of mitophagy on DKD. BNIP3, BCL2-Interacting Protein 3; BNIP3L, BNIP3-Like; MQC, Mitochondrial quality control; Nrf1/2, Nuclear Respiratory Factor 1/2; OPA1, optic atrophy protein 1; PGC-1α, peroxisome proliferator-activated receptor-γ coactivator 1α; PPARs, Peroxisome Proliferator Activated Receptors; TFAM, mitochondrial transcription factor A; Rheb1, Ras homolog. The following images were created by BioGDP.com (145).

MQC is a highly dynamic regulatory mechanism that maintains cellular function and homeostasis. It typically eliminates damaged mitochondria and synthesizes new intact mitochondria through processes such as mitochondrial biogenesis, mitochondrial dynamics, and mitophagy. MQC plays a critical role in regulating energy metabolism, maintaining calcium homeostasis and redox balance, and modulating the cell cycle and programmed cell death (PCD) (12, 22). The kidneys are the primary oxygen-consuming organs in the body, requiring substantial amounts of ATP produced through mitochondrial OXPHOS to sustain their function (23). Research indicates that regulating MQC can alleviate kidney-related diseases, such as renal cell senescence and fibrosis (24), mitigate acute kidney injury (25), and reduce diabetic kidney disease (26). Therefore, mitochondrial quality control acts as an adaptive response under various physiological or pathological conditions to ensure the proper morphology, quantity, and function of mitochondria, thereby maintaining optimal renal function. The mechanism diagram of MQC in DKD is shown in **Figure 1**.

### 2.1 Mitochondrial biogenesis in DKD

Mitochondrial biogenesis is a complex process that enhances mitochondrial mass and copy number to meet energy demands by generating new mitochondria through the growth and division of existing ones (13). This intricate cellular process is regulated by a network of transcription factors and coactivators. Among them, the sirtuin 1/peroxisome proliferator-activated receptor-γ coactivator 1α (SIRT1/PGC-1α) signaling pathway and its downstream targets, nuclear factor erythroid 2-related factor 1 (NRF1), nuclear factor erythroid 2-related factor 2 (NRF2), and mitochondrial transcription factor A (TFAM), play crucial roles in driving mitochondrial biogenesis (22, 27). Research suggests that PGC-1α can be activated by various post-translational modifications, such as phosphorylation or deacetylation. Activated PGC-1α translocates to the nucleus and activates NRF1 and NRF2, subsequently inducing the transcription of nuclear-encoded respiratory chain components and TFAM. This process promotes mitochondrial protein synthesis, mitochondrial DNA replication and transcription, ultimately enhancing mitochondrial biogenesis (28, 29).

Recent studies have shown that GLIS1 (Gli-like transcription factor 1), as a metabolic regulatory transcription factor, can modulate MQC by interacting with PGC-1α, thereby alleviating renal cell senescence and fibrosis (24). DKD is a major cause of CKD and ESRD, with pathological manifestations including glomerulosclerosis, tubular injury, and renal fibrosis (30). Research has found that the absence of immune response gene 1 (IRG1) exacerbates renal tubular damage in diabetic mice. Ultrastructural analysis of mouse kidneys revealed marked mitochondrial dysfunction, characterized by swelling, deformation, and disruption or loss of cristae. Treatment with the itaconic acid derivative 4-octyl itaconate (4-OI) alleviated tubular injury and improved renal function in db/db mice. Mechanistically, 4-OI mitigates tubular damage in diabetic nephropathy mice by activating the NRF2/PGC-1α signaling axis, thereby promoting mitochondrial biogenesis (31). Additionally, studies indicate that renal tubular epithelial injury constitutes an early event in DKD. Adiponectin facilitates the repair of these injured cells by enhancing mitochondrial biogenesis, primarily via the AdipoR1/CREB/PGC-1α/TFAM signaling pathway. The above findings emphasize the strong link between mitochondrial biogenesis and DKD, suggesting that modulating mitochondrial biogenesis could effectively reduce renal injury.

### 2.2 Mitochondrial dynamics in DKD

Mitochondrial dynamics refer to the changes in the morphology, number, and distribution of mitochondria within eukaryotic cells and primarily encompass two processes: fusion and fission (32). Fusion is the merging of two or more mitochondria into larger organelles, enabling the mixing and exchange of mitochondrial contents. Fission is the process of constricting and cleaving a mitochondrion to generate smaller organelles, enabling the elimination of damaged or dysfunctional mitochondria to promote MQC and facilitate apoptosis (33, 34). Mitochondrial fusion and fission are critically important for maintaining mitochondrial integrity and homeostasis (35).

Mitochondrial fusion is a complex process mediated by three membrane-associated GTPases: mitofusin 1 (MFN1, located on human chromosome 3q26), mitofusin 2 (MFN2, located on human chromosome 1p36), and optic atrophy 1 (OPA1, located on human chromosome 3q29).These proteins link two mitochondria at the interface between the outer and inner membranes (36). OPA1 anchors to the inner mitochondrial membrane (IMM) via its unique N-terminal transmembrane domain. Upon GTP binding, it undergoes conformational changes that facilitate inner membrane fusion, which is crucial for maintaining mitochondrial morphology and function (37, 38). MFN1 and MFN2, which share similar structures and functions, function as bridges to promote outer mitochondrial membrane (OMM) fusion. Through their α-helical regions, MFNs form homodimers or heterodimers (39). OPA1 is essential for maintaining mitochondrial morphology and function in podocytes. Under hyperglycemic (HG) conditions, OPA1 expression in cultured podocytes is reduced. Moreover, as DKD progresses, OPA1 expression in glomeruli decreases progressively. The peptide SS31 protects podocytes by inhibiting OMA1-mediated OPA1 cleavage, thereby slowing DKD progression (40). Furthermore, kaempferol improves renal function and ameliorates pathological injury in diabetic nephropathy rats. This effect is achieved through the regulation of mitochondrial dynamics and mitophagy, leading to enhanced mitochondrial fusion and restored mitophagy function (41).

Mitochondrial fission is primarily regulated by dynamin-related protein 1 (Drp1) and its receptors, including mitochondrial fission factor (MFF) and fission 1 protein (Fis1). As a key executor of mitochondrial fission, Drp1 translocates from the cytoplasm to the outer mitochondrial membrane (OMM) in response to cellular signals initiating fission (42). This process is largely mediated by Fis1, which interacts with the OMM through its C-terminal transmembrane domain, localizing to the membrane and recruiting Drp1 (43). Once recruited, Drp1 oligomerizes through interactions with other Drp1 molecules on the mitochondrial surface, forming ring-like or helical structures. These assemblies then utilize GTP hydrolysis to drive mitochondrial constriction and fission (44, 45). Research indicates that MFF is similarly localized to and functions like FIS1, as it is capable of recruiting the oligomeric form of Drp1 and enhancing its GTPase activity (46). Reports indicate that DKD involves excessive mitochondrial fission and increased expression of specificity protein 1 (SP1) and phosphoglycerate mutase family member 5 (PGAM5). Empagliflozin (Empa) alleviates mitochondrial fission and improves DKD by activating the AMPK/SP1/PGAM5 signaling pathway (47). Endothelial cell injury is regarded as a critical initiating event in the development of DKD. Evidence suggests that albuminuria resulting from endothelial dysfunction exerts cytotoxic effects on podocytes, a process closely linked to disturbances in mitochondrial dynamics. Modulation of the mitochondrial fission protein Drp1 can ameliorate such dysregulated dynamics, thereby effectively mitigating albuminuria-induced podocyte injury in DKD (48). In summary, the balance between mitochondrial fusion and fission is essential for maintaining renal cellular homeostasis. Targeting mitochondrial dynamics has therefore emerged as a promising therapeutic strategy to slow DKD progression, offering a valuable direction for future research and intervention.

### 2.3 Mitophagy in DKD

In mammalian cells, autophagy is classified into three main types: macroautophagy, microautophagy, and chaperone-mediated autophagy. Mitophagy, a selective form of autophagy, involves the engulfment and degradation of damaged mitochondria, thereby maintaining mitochondrial and cellular homeostasis (49, 50). Mitophagy can be classified into two distinct pathways: ubiquitin-dependent mitophagy and ubiquitin-independent (receptor-mediated) mitophagy. The PTEN-induced putative kinase 1 (PINK1)/Parkin pathway, which functions as the ubiquitin-dependent pathway, is the most extensively studied (51). PTEN-induced kinase 1 (PINK1) is a kinase encoded by the PARK6 gene that serves as a sensor for mitochondrial damage. Following mitochondrial injury, PINK1 accumulates on the mitochondrial surface, where it phosphorylates proteins on the outer mitochondrial membrane (OMM) and the E3 ubiquitin ligase Parkin. This leads to the recruitment and activation of Parkin on the OMM (52). Once activated, Parkin ubiquitinates numerous OMM proteins. These ubiquitinated proteins are further phosphorylated by PINK1, creating a positive feedback loop that recruits additional Parkin and amplifies ubiquitin chain formation. Ultimately, the ubiquitin chains on the mitochondria are recognized by autophagy adaptors, leading to the sequestration and degradation of the damaged organelle via mitophagy (53).

Myricetin (Myr), a polyphenolic extract from *Rubus suavissimus* S. Lee (RS), has been reported to play a crucial role in mitigating cellular damage in DKD models by regulating mitochondrial function. Myr promotes XBP1 expression, which in turn activates the PI3K/Akt and PINK1/Parkin pathways, thereby enhancing autophagic flux and providing renal protection in DKD (54). These findings underscore the therapeutic potential of targeting the PINK1-Parkin signaling axis to reduce renal injury and maintain mitochondrial homeostasis. In ubiquitin-independent pathways, mitophagy is mediated by specific receptor proteins such as BCL2-Interacting Protein 3 (BNIP3), BNIP3-Like (BNIP3L), FUNDC1, and AMBRA1, which directly recruit LC3 to promote mitochondrial engulfment (55). To investigate the mechanism of regulatory T cells (Tregs) in DKD podocyte injury, GUO et al. employed sequencing and cross-sectional analysis. They identified the TNC/TLR4/SRC/FUNDC1 pathway as crucial for the function of Treg-derived exosomes (Treg-Exos). By engineering Treg-Exos to display RGD peptides on their surface, they enabled targeted binding to integrins on podocytes, facilitating the delivery of miR-218-5p. This strategy enhanced mitophagy, reduced apoptosis, and alleviated podocyte injury (56). Additionally, the circadian rhythm gene brain and muscle ARNT-like 1 (BMAL1) was found to alleviate renal ischemia–reperfusion injury (I/RI) in diabetic mice, primarily through promoting HIF-1α/BNIP3-mediated mitophagy, which exerts protective effects against I/RI in diabetic kidneys (57). Collectively, these studies highlight the protective role of mitophagy in maintaining renal health.

## 3 Epigenetics and post-translational modifications

Epigenetics refers to heritable changes in gene expression without altering the DNA sequence. It involves the control of chromatin-associated proteins and reversible chemical modifications of DNA and histones, which collectively regulate chromatin structure to maintain transcriptional programs. These mechanisms modulate gene expression by controlling DNA accessibility through chromatin architecture and transcriptional regulation (58, 59). The primary epigenetic mechanisms include DNA methylation, histone modifications, chromatin remodeling, and non-coding RNA (ncRNA) regulation, among which DNA methylation, histone modifications, and ncRNA regulation are the most prevalent (60, 61). Epigenetic machinery is categorized into four functional classes: “writers,” “erasers,” “readers,” and “remodelers.” Writers are enzymes that add covalent modifications to DNA and histones, whereas erasers remove these modifications. Readers recognize specific marks through dedicated domains to mediate downstream effects, and remodelers regulate chromatin architecture and compaction. In essence, writers deposit modifications that alter nucleosomal interactions and serve as binding platforms for readers. Erasers catalyze the removal of these modifications, enabling dynamic and reversible regulation (62, 63). Histones undergo a wide array of PTMs, including phosphorylation, ubiquitination, SUMOylation, acetylation, methylation, ADP-ribosylation, and others such as palmitoylation, lipoylation, glycosylation, isoprenylation, cholesterylation, myristoylation, glutathionylation, sulfation, citrullination, and S-nitrosylation. The most characteristic modifications occur on amino acids within the flexible N-terminal tails of histones, which extend from the nucleosome core (15, 64). Although proteins are synthesized from only 20 standard amino acids, their chemical and functional diversity is vastly expanded through PTMs of various residues (65). Epigenetic modifications and PTMs respectively regulate gene expression at the transcriptional and post-translational levels, thereby modulating protein activity, function, and stability. These modifications are essential for maintaining tissue-specific gene expression profiles, as well as normal cellular development and function (61). Emerging evidence suggests that MQC contributes to the epigenetic regulation of DKD, an area that has garnered considerable interest in nephrology research.

## 4 Epigenetic and posttranslational modifications regulating MQC in DKD

**Figure 2:**
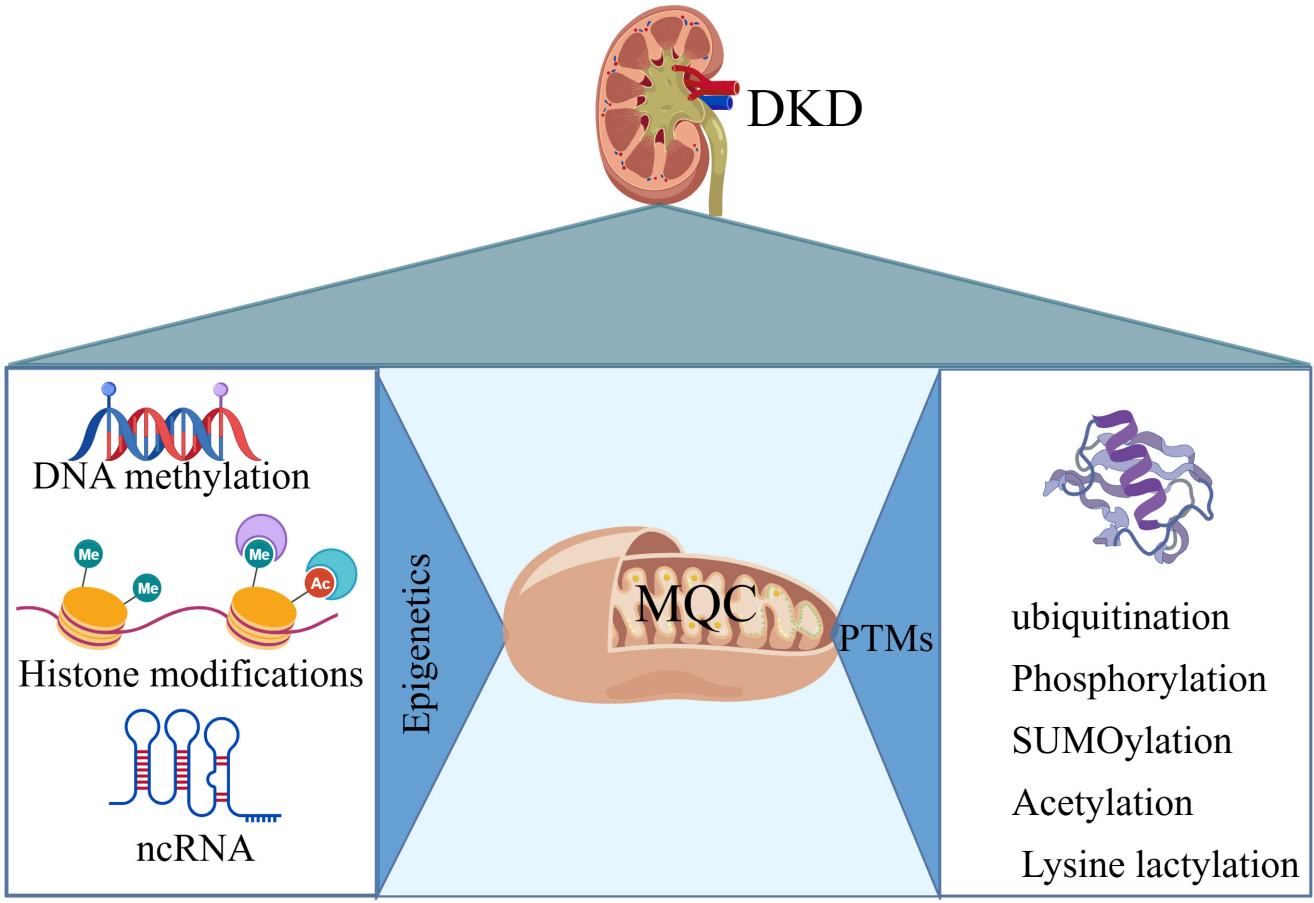
The regulation of mitochondrial quality control by epigenetic factors and post-translational modifications in diabetic nephropathy. Epigenetic modifications primarily include DNA methylation, histone modifications, and non-coding RNAs; post-translational modifications primarily include ubiquitination, phosphorylation, SUMOylation, acetylation, and lysine lactylation.

Epigenetic modifications and PTMs play critical roles in regulating MQC in DKD. These mechanisms interact at multiple levels to orchestrate mitochondrial dysfunction. The crosstalk among them is summarized in **Figure 2**.

### 4.1 DNA methylation-mediated regulation of MQC in DKD

**Figure 3:**
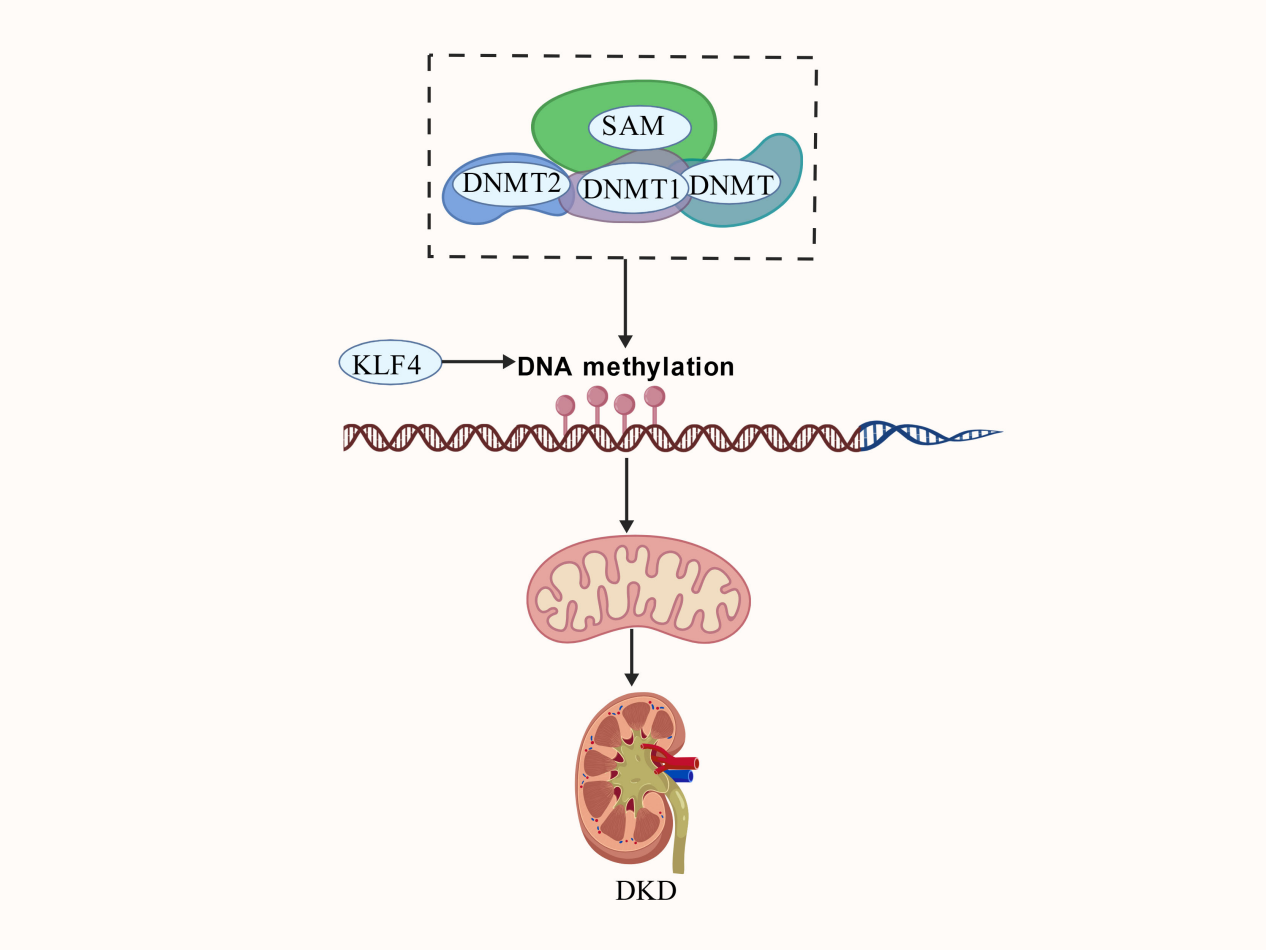
The mechanism of action between DNA methylation and mitochondrial quality control in DKD. DNMT1/2, DNA methyltransferase 1/2; KLF4, Krüppel-like factor 4; SAM, S Adenosyl-L-Methionine.

DNA methylation is a covalent modification that plays a fundamental role in gene regulation and cellular identity in mammals. During this process, DNA methyltransferases (DNMTs) transfer a methyl group from S-adenosylmethionine (SAM) to the carbon-5 position of the cytosine ring (5mC) within the cytosine-guanine (CpG) dinucleotide (66). Typically, CpG islands typically remain unmethylated and serve as binding sites for transcription factors (67). The reaction is catalyzed by DNMTs, primarily members of the DNMT1 and DNMT3 families (DNMT3a and DNMT3b), which use SAM as a methyl donor (68). Recent research has revealed that DNMT1 also regulates mitochondrial function via mediating m^5^C RNA methylation. Specifically, DNMT1 binds to mRNA transcripts and promotes 5-methylcytosine (m5C) modification by recruiting the NOP2/Sun RNA methyltransferase 2 (NSUN2). Aberrant RNA m5C modifications are associated with mitochondrial dysfunction (69). Notably, studies indicate that the regulation of mitochondrial inner membrane potential (ΔΨm) is mechanistically linked to nuclear DNA methylation. Meanwhile, mitochondrial membrane hyperpolarization affects DNA methylation and gene expression primarily through phospholipid remodeling, rather than via redox or metabolic changes (70). In contrast, demethylation is initiated by the oxidation of 5-methylcytosine by TET enzymes (ten-eleven translocation enzymes), converting it into 5-hydroxymethylcytosine (71). This process promotes the binding of transcription factors (TFs) to the newly unmethylated CpG sites, thereby upregulating target gene expression. Additionally, DNA methylation marks are recognized by the methyl-CpG binding domain (MBD) protein family, which binds to 5-methylcytosine at CpG sites and modifies the surrounding chromatin structure (72). Emerging studies suggest that DNA methylation modification regulates MQC in DKD (**Figure 3**).

Insulin resistance increases the glomerular filtration of albumin and overloads tubular reabsorption capacity, thereby impairing albumin reuptake (68). Among various epigenetic modifications, altered DNA methylation of genes involved in insulin signaling has been recognized as a prominent epigenetic marker in multiple DKD cohorts worldwide, with insulin resistance itself being an associated risk factor for such methylation changes (73). In addition, studies indicate that mitochondrial DNA methylation can provide protection against renal tubular epithelial cell (RTEC) damage by reducing the production of reactive oxygen species (mtROS) (74). The zinc-finger transcription factor Krüppel-like factor 4 (KLF4), a member of the SP/KLF family, has been linked to DKD pathogenesis. Research shows that berberine improves glycemic control and renal function in a mouse model of diabetic nephropathy by inhibiting the expression of DNA methyltransferases DNMT1 and DNMT3a. This inhibition significantly reduces promoter methylation of the KLF4 gene, promotes mitochondrial structural maintenance, and prevents ferroptosis, collectively protecting renal architecture and inhibiting fibrosis (75). Above all, these findings underscore that DNA methylation is involved in DKD progression and plays a key role in delaying or ameliorating DKD by regulating mitochondrial homeostasis. However, the mechanism by which DNA methylation modulates MQC in DKD remains poorly understood. Further research is needed to elucidate the regulatory mechanisms among these three components.

### 4.2 Post-translational modifications regulating MQC in DKD

#### 4.2.1 Ubiquitin-mediated regulation of MQC in DKD

Ubiquitin (Ub) is a 76−amino acid protein containing seven lysine residues that can serve as attachment sites for further ubiquitination, thereby forming distinct polyubiquitin chains. These chains are recognized by various effector proteins (76). Protein ubiquitination proceeds through a three-step enzymatic cascade involving ubiquitin-activating enzymes (E1), ubiquitin-conjugating enzymes (E2), and ubiquitin-protein ligases (E3). E3 ligases selectively recognize substrate proteins and recruit the Ub-E2 complex to catalyze the transfer of Ub from E2 to the substrate, typically via an isopeptide bond (77). Deubiquitinating enzymes (DUBs) reverse this modification by removing ubiquitin chains, thereby regulating substrate stability and function (78). The dynamic interplay between ubiquitinating enzymes and DUBs plays a critical role in regulating nearly all aspects of cellular activity. Emerging evidence indicates that the balance of ubiquitination and deubiquitination regulates MQC in DKD, suggesting that specific modulators targeting these pathways may offer therapeutic potential by fine−tuning the ubiquitination of MQC factors.

Indole-3-propanoic acid (IPA), a gut microbiota-derived metabolite of dietary tryptophan, has been shown to increase SIRT1 levels by inhibiting its ubiquitin-dependent degradation. This upregulation activates the SIRT1/PGC-1α signaling pathway, promotes mitochondrial biogenesis, and reduces oxidative stress in glomerular endothelial cells, thereby mitigating the progression of diabetic kidney disease (79). Additionally, the E3 ubiquitin ligase retinoblastoma-binding protein 6 (RBBP6) has been identified as a negative regulator of estrogen-related receptor alpha (ERRα), targeting it for K48-linked polyubiquitination and degradation. ERRα, an orphan nuclear receptor highly expressed in renal PTCs, is destabilized by this pathway, which exacerbates mitochondrial damage in DKD models. Conversely, inhibiting RBBP6 or overexpressing ERRα reduces mitochondrial damage, preserves mitochondrial integrity, and slows DKD progression in diabetic mouse models (80). The tripartite motif-containing (TRIM) family of E3 ubiquitin ligases regulates protein quality control via the ubiquitin-proteasome system. Research indicates that N6-methyladenosine (m^6^A) modification of TRIM22 mRNA, mediated by Wilms tumor 1-associating protein (WTAP) and insulin-like growth factor 2 mRNA-binding protein 1 (IGF2BP1), accelerates DKD progression by inducing mitochondrial dysfunction. This effect occurs through TRIM22-mediated ubiquitination of OPA1, which inhibits mitochondrial fusion (81). Furthermore, researchers have discovered that the E3 ubiquitin ligase Cullin3 (CUL3) induces K63-linked ubiquitination of mitochondrial ribosomal protein L12 (MRPL12) in renal tubular epithelial cells, impairing mitochondrial biogenesis. This disruption exacerbates high glucose-induced mitochondrial dysfunction and accelerates DKD progression (82).

#### 4.2.2 Phosphorylation-mediated regulation of MQC in DKD

Phosphorylation is the most prevalent post-translational modification of proteins, affecting approximately 30% of the human proteome and regulating nearly all cellular processes (83). The reaction primarily targets serine (Ser), threonine (Thr), and tyrosine (Tyr) residues, with serine phosphorylation accounting for more than 75% of all cases (84). Histone phosphorylation occurs predominantly on these residues within the N- and C-terminal tails, where it serves as a key regulator of gene expression. Mechanistically, the addition of negatively charged phosphate groups neutralizes the positive charge of histone tails, loosening chromatin compaction and facilitating transcription (85). Accumulating evidence indicates that phosphorylation also plays a regulatory role in MQC during DKD pathogenesis.

Studies have shown that deficiency of insulin-like growth factor-binding protein 2 (IGFBP2) slows DKD progression, reduces proteinuria and renal pathology, and alleviates podocyte apoptosis. Mechanistically, IGFBP2 binds to integrin α5 (ITGA5) on podocytes, triggering phosphorylation of focal adhesion kinase (FAK). FAK activation promotes mitochondrial oxidative damage and apoptosis in podocytes, thereby accelerating DKD. Conversely, inhibiting the ITGA5/FAK pathway mitigates IGFBP2-induced mitochondrial ROS production and podocyte injury (86). A-kinase-associated protein 1 (AKAP1) is an anchoring protein located on the outer mitochondrial membrane (OMM) that is extensively involved in regulating functions related to mitochondrial metabolism and dynamics (87). JunFeng et al. demonstrated that AKAP1 recruits PKC to phosphorylate La-related protein 1 (LARP1), leading to decreased expression of mitochondrial transcription factor A (TFAM) and impaired mitochondrial DNA replication. This cascade ultimately contributes to mitochondrial dysfunction and podocyte injury in DKD (88). Additionally, cyclin-dependent kinase 5 (Cdk5), a proline-directed serine/threonine kinase, modulates mitochondrial function via Sirt1 phosphorylation to influence podocyte injury in DKD. Specifically, Cdk5 phosphorylates Sirt1 at Ser47, compromising mitochondrial function and promoting podocyte damage. Thus, inhibiting Cdk5 represents a potential therapeutic strategy for mitigating podocyte injury and slowing DKD progression (89, 90).

#### 4.2.3 Acetylation-mediated regulation of MQC in DKD

Protein acetylation is a prevalent and dynamic post-translational modification mediated by acetyltransferases (HATs). Histone acetylation involves the addition of an acetyl group to lysine residues within histone N-terminal tails (91). Both histone and non−histone acetylation regulate a broad spectrum of cellular processes, including enzyme activity, chromatin architecture, transcription, protein localization, protein−protein interactions, and metabolism (92). Accumulating evidence suggests that protein acetylation plays a key role in mitochondrial-related functions, such as mitochondrial dynamics and the nuclear localization of mitochondrial TCA cycle enzymes (93, 94). Emerging research indicates that acetylation is involved in regulating MQC in DKD.

SIRT3 is a crucial mitochondrial deacetylase that maintains mitochondrial homeostasis. Studies show that the novel coumarin derivative SZC−6 activates SIRT3, which directly deacetylates Foxo3a at Lys271. This promotes FOXO3a nuclear accumulation in renal tubular cells, upregulates the antioxidant enzyme MnSOD, restores mitochondrial function and tubular homeostasis, and alleviates DKD injury (95). Furthermore, the SIRT3/MPC2 axis has been identified as a potential therapeutic target in DKD. Specifically, SIRT3 deacetylates mitochondrial pyruvate carrier 2 (MPC2) at lysines 19 and 27 (K19/K27) on the inner mitochondrial membrane. Downregulation of SIRT3 in DKD podocytes exacerbates MPC2 acetylation, leading to pyruvate accumulation, mitochondrial dysfunction, and podocyte injury, thereby driving DKD progression (96). Rheb1, a homolog of Rheb, regulates mitochondrial biogenesis and material metabolism in conjunction with mTORC1 (97). Qingmiao et al. demonstrated that Rheb1 deficiency promotes acetylation of Atp5f1c in an mTORC1−independent manner, which increases ROS production and PDH phosphorylation while reducing ATP levels in DKD. These changes result in mitochondrial dysfunction and accelerated podocyte senescence (98).

#### 4.2.4 SUMOylation-mediated regulation of MQC in DKD

SUMOylation is a post-translational modification that involves the covalent attachment of small ubiquitin-like modifier (SUMO) proteins to specific lysine residues on target substrates, thereby critically regulating diverse cellular processes (99). As members of the ubiquitin-like protein family, SUMO proteins share structural similarity with ubiquitin and are conjugated to target proteins through an enzymatic cascade that results in the formation of an isopeptide bond between the C-terminal glycine of SUMO and the ϵ-amino group of a lysine residue on the substrate (100). This process is mediated by a three−enzyme cascade: the heterodimeric SUMO-activating E1 enzyme (SAE1−SAE2/UBA2), the single E2 conjugating enzyme (UBC9), and a limited number of E3 ligases (101). During SUMOylation, the E1 enzyme first activates SUMO in an ATP−dependent manner. The activated SUMO is then transferred to UBC9 (E2) and, with the assistance of an E3 ligase, is finally conjugated to a specific lysine residue on the substrate (102). As a dynamic and reversible modification, SUMOylation regulates diverse cellular functions and is essential for maintaining cellular homeostasis and organismal health. Asprosin (ASP), a peptide hormone primarily secreted by white adipose tissue, impairs mitochondrial dynamic homeostasis in renal TECs of DKD models. Specifically, ASP promotes excessive SUMO1 modification of DRP1, which stabilizes Drp1 by reducing its degradation and disrupts mitochondrial dynamics. This dysregulation contributes to TEC injury and phenotypic transition. In this pathway, the deSUMOylating protease SENP1 and the SUMO E3 ligase PIAS1 are critical regulators and represent potential therapeutic targets (103). Furthermore, RNA−binding motif protein X−linked (RBMX) exacerbates DKD by promoting the exosomal export of protective miRNAs from TECs. Mechanistically, RBMX undergoes self−SUMOylation with SUMO3 at lysine 80, enabling it to bind and package miRNAs into exosomes. This removal prevents miRNAs from suppressing the mitochondrial regulator CERS6, thereby worsening mitochondrial damage (104).

#### 4.2.5 Lactylation-mediated regulation of MQC in DKD

Lactic acid, traditionally regarded as a metabolic waste product, is the end product of glycolysis. In the 1920s, Otto Warburg observed that cancer cells primarily generate energy through glycolysis and increase lactic acid production even in aerobic environments, rather than relying on the oxidative phosphorylation process commonly used by normal cells (105). This association with tumor metabolism has since drawn considerable scientific interest to lactate’s biological roles. In 2019, a landmark study in Nature identified a novel form of post−translational modification termed lysine lactylation (Kla), which involves the covalent attachment of a lactate moiety to lysine residues, initially characterized on nuclear histones (106). Multiple lactylation sites have been mapped across histones H3, H4, H2A, and H2B. Notably, the landscape of histone lactylation sites varies between species, and growing evidence indicates that non−histone proteins can also undergo lactylation (107). Emerging research now suggests that protein lactylation may regulate MQC and influence the pathogenesis of kidney diseases. Acyl−CoA synthetase family member 2 (ACSF2) catalyzes the initial activation of fatty acids via formation of a thioester with coenzyme A (108). Studies reveal a marked increase in lysine lactylation in the kidneys of diabetic patients and db/db mice, with most lactylated proteins localized to mitochondria and implicated in metabolic pathways. Specifically, lactylation of ACSF2 at lysine 182 (K182la) contributes to elevated ROS accumulation and mitochondrial damage in high glucose−treated HK−2 cells (109). These findings suggest that lactylation of mitochondrial proteins, including ACSF2, may play a critical role in the pathogenesis of DKD.

**Figure 4:**
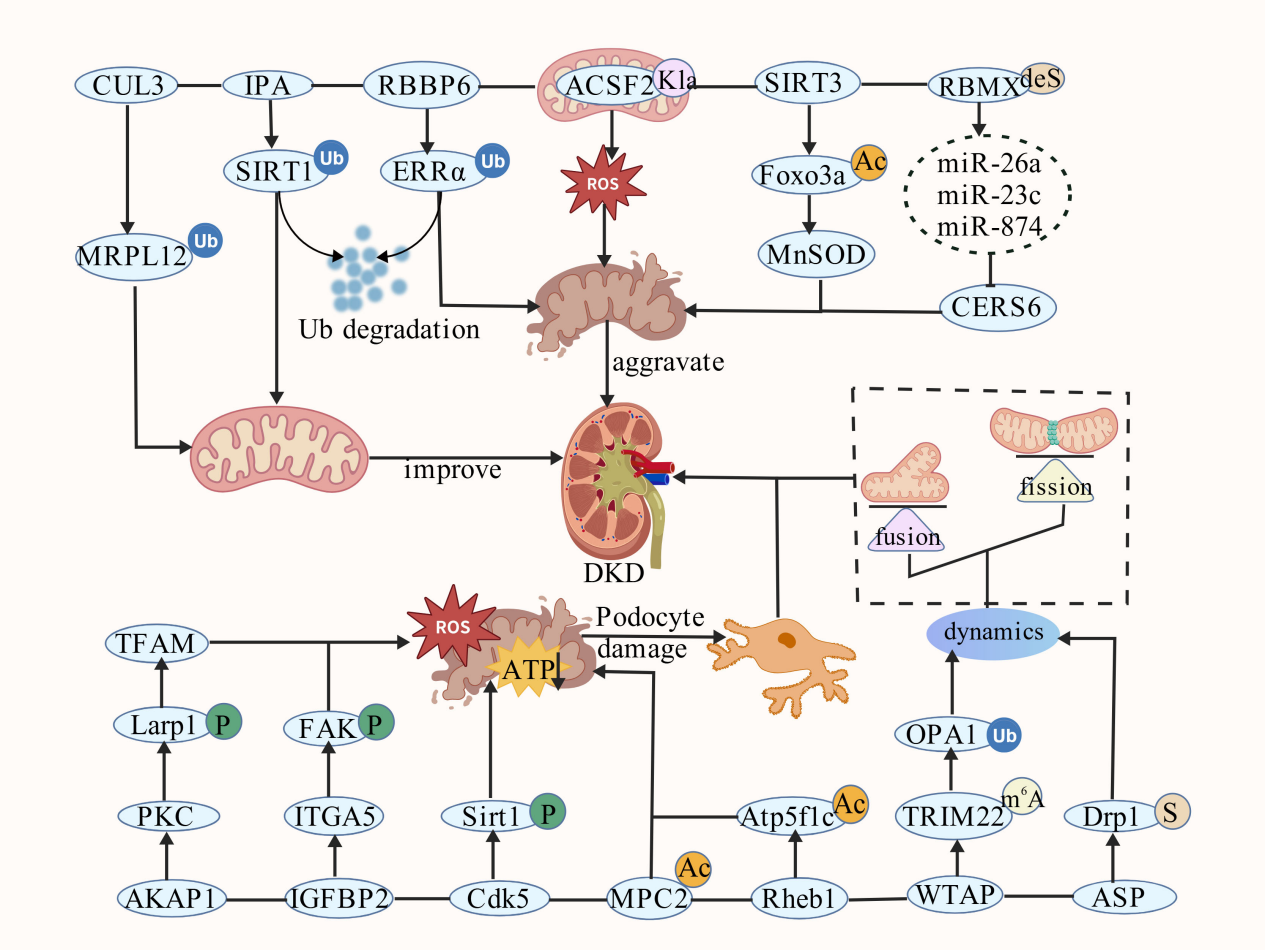
The mechanism of interaction between posttranslational modifications and mitochondrial quality control in DKD. ACSF2, Acyl-CoA synthetase family member 2; AKAP1, A-kinase anchoring protein 1; Ac, Acetylation; ASP, Asprosin; CERS6, ceramide synthase 6; Cdk5, Cyclin-dependent kinase 5; CUL3, Cullin3; deS, deSUMOylation; IGFBP2, Insulin-like growth factor-binding protein 2; IPA, indole-3-propionic acid; ITGA5, Integrin α 5; Kla, Lysine lactylation; m6A, N6 methyladenosine; MPC2, Mitochondrial pyruvate carrier 2; MRPL12, mitochondrial ribosomal protein L12; P, Phosphorylation; RBBP6, retinoblastoma binding protein 6; S, SUMOylation; Ub, Ubiquitination..

In summary, PTMs form an interconnected network regulating MQC in DKD. Ubiquitination and phosphorylation provide rapid control of protein stability and signaling, while acetylation (especially SIRT3-mediated) modulates mitochondrial function and antioxidant defense. SUMOylation and lactylation further link metabolic stress to mitochondrial dynamics and ROS production. These modifications often crosstalk with each other and with upstream epigenetic changes, amplifying mitochondrial dysfunction under hyperglycemia. (**Figure 4** and **Table 1**).

**Table 1:** Posttranslational modifications of MQC in DKD.

Modification	Model	Mechanism of action	Regulation of MQC	Effect	Ref
Ubiquitination	*In vivo*: Male C57BL/6 mice *In vitro*: Primary glomerular endothelial cells (GECs)	SIRT1/PGC-1α	Mitochondrial biogenesis	IPA inhibits ubiquitination-mediated SIRT1 degradation, thereby activating the SIRT1/PGC-1α pathway and promoting mitochondrial biogenesis to alleviate DKD progression.	(79)
	*In vivo*: Male C57BL/6N mice *In vitro*:HK-2 cells	RBBP6-ERRα	Mitochondrial biogenesis	RBBP6 promotes K48-linked polyubiquitination of ERRα on the K100 residue, thereby influencing mitochondrial damage in proximal tubule cells of DKD.	(80)
	*In vivo*: Male C57BL/KsJ *In vitro*: HK-2 cells	WTAP/IGF2BP1-TRIM22-OPA1	Mitochondrial dynamics	WTAP-mediated m^6^A modification of TRIM22 promotes DKD by inducing mitochondrial dysfunction via ubiquitination of OPA1 at K228.	(81)
	*In vitro*: HK-2 cells	CUL3/MRPL12	Mitochondrial biogenesis	CUL3 directly interacts with MRPL12, inducing K63-linked ubiquitination of MRPL12, which leads to mitochondrial biogenesis dysfunction in renal tubular epithelial cells.	(82)
Phosphorylation	*In vivo*: the male IGFBP2 null and wild type (WT) FVB mice *In vitro*: The human podocyte cell line	IGFBP2/ITGA5/FAK/ROS	Mitochondrial ROS generation	IGFBP2 binds to podocyte ITGA5 to activate FAK phosphorylation, triggering mitochondrial oxidative damage and apoptosis, thereby exacerbating DKD.	(86)
	*In vivo*: male Sprague-Dawley rats *In vitro*: Conditionally immortalized human podocytes	AKAP1/PKC/Larp1/TFAM	Mitochondrial biogenesis	AKAP1 induces PKC-mediated Larp1 phosphorylation, reducing TFAM expression and exacerbating mitochondrial dysfunction and podocyte injury in DKD.	(88)
	*In vivo*: male CD1 mice male C57BLKS/J db/db mice *In vitro*: HPCs	Cdk5/Sirt1	Mitochondrial dynamics	Cdk5 phosphorylates Sirt1 at S47, causing mitochondrial dysfunction and podocyte injury, thereby contributing to the progression of DKD.	(90)
Acetylation	*In vivo*: Male C57BL/6 J mice *In vitro*: HK-2 cells	SIRT3-Foxo3a	Mitochondrial dynamics and mitochondrial ROS generation	SZC-6 deacetylates Foxo3a at Lys 271 to upregulate MnSOD activity, thereby suppressing oxidative stress and mitochondrial dysfunction to alleviate DKD.	(95)
	*In vivo*: male Sprague–Dawley rats *In vitro*: Conditionally immortalized human podocytes	SIRT3/MPC2	Mitochondrial ROS generation, decrease mitochondrial membrane potential and ATP levels	SIRT3 overexpression reduces MPC2 acetylation at K19/K27, thereby mitigating mitochondrial damage and podocyte apoptosis to alleviate DKD.	(96)
	*In vivo*: C57BL/6 J mice *In vitro*: podocyte cell line	Rheb1/Atp5f1c	Mitochondrial ROS generation, decrease mitochondrial membrane potential and ATP levels	Rheb1 deficiency promotes mTORC1-independent Atp5f1c acetylation, driving mitochondrial dysfunction and podocyte senescence.	(98)
SUMOylation	*In vivo*: C57BL/6male mice *In vitro*: HK-2 cells	SENP1/PIAS1/Drp-SUMO1	Mitochondrial dynamics	Asprosin-induced Drp1 hyper-SUMoylation impairs mitochondrial dynamics, exacerbating tubular injury and phenotypic transition in DKD.	(103)
	*In vivo*: C57BL6/J mice and db/db mice *In vitro*: HK-2 cells	RBMX/CERS6	Increase mitochondrial ROS, decrease mitochondrial membrane respiratory chain complex proteins and ATP levels	K80 de-SUMOylation of RBMX regulates exosomal cargo sorting to promote mitochondrial damage and renal fibrosis in DKD.	(104)
Lactylation	*In vivo*: male db/db Mice C57BL/KsJ *In vitro*: HK-2 cells	ACSF2	Mitochondrial dynamics	ACSF2-mediated K182la modification promotes ROS accumulation and mitochondrial damage in high-glucose-treated HK-2 cells.	(109)

### 4.3 Non-coding RNA-mediated regulation of MQC in DKD

**Figure 5:**
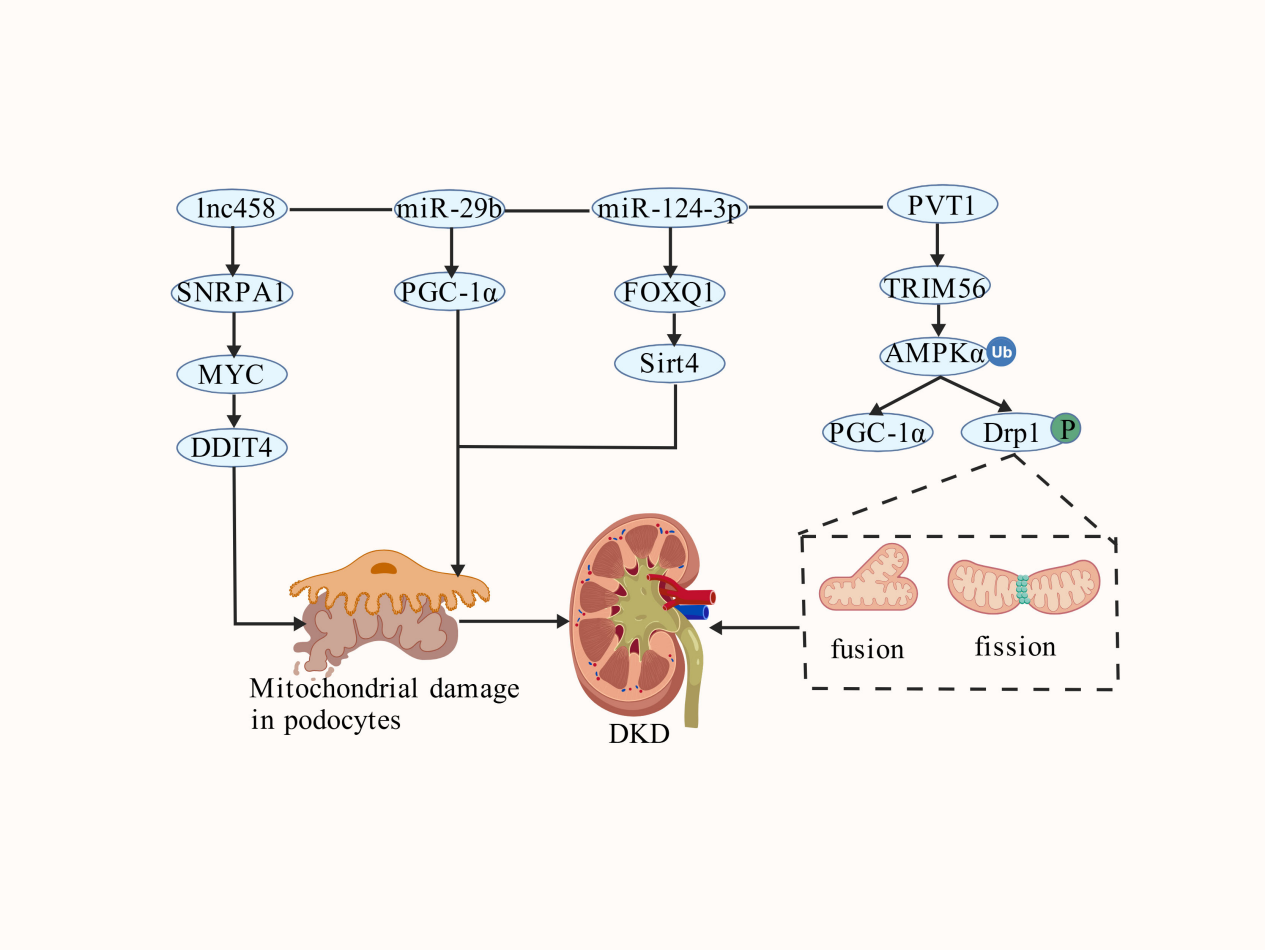
The mechanism of interaction between non-coding RNA and mitochondrial quality control in DKD. DDIT4, DNA damage-inducible transcript 4; FOXQ1, Forkhead box Q1, lnc458, lncRNA ENST00000458139; PVT1, LncRNA plasmacytoma variant translocation 1; SNRPA1, Small nuclear ribonucleoprotein polypeptide A; Sirt4, Sirtuin 4, TRIM56, tripartite motif 56.

Approximately 75% of the human genome is transcribed into RNA, yet only about 3% encodes proteins (110). Non-coding RNAs (ncRNAs) are ubiquitous transcriptional products that, despite not encoding proteins, play crucial regulatory roles in gene expression and protein function (111). Advances in high−throughput sequencing have unveiled a diverse landscape of ncRNAs, including microRNAs (miRNAs), long non−coding RNAs (lncRNAs), circular RNAs (circRNAs), small interfering RNAs (siRNAs), small nucleolar RNAs (snoRNAs), ribosomal RNAs (rRNAs), and transfer RNAs (tRNAs). Among these, miRNAs, lncRNAs, and circRNAs are prominently involved in post−transcriptional regulation (112, 113). Emerging evidence highlights that ncRNAs—particularly miRNAs, lncRNAs, and circRNAs—significantly influence MQC by modulating molecular pathways that govern mitochondrial homeostasis in kidney diseases (**Figure 5** and **Table 2**).

**Table 2:** Non-coding RNA modifications of MQC in DKD.

Modification	Model	Mechanism of action	Regulation of MQC	Effect	Ref
Non-coding RNA	*In vivo*: db/db mice Male BALB/c mice *In vitro*: MPC5 293T	miR-29b/PGC-1α	Mitochondrial biogenesis	MiR-29b targets PGC-1α to inhibit fatty acid oxidation and mitochondrial biogenesis, driving lipid accumulation and mitochondrial dysfunction.	(116)
	*In vivo*: Male C57BL/6 mice *In vitro*: podocyte cell line	LncRNA PVT1 /TRIM5	Mitochondrial biogenesis and mitochondrial fusion	PVT1 interacts with TRIM56 to regulate AMPKα ubiquitination, driving abnormal mitochondrial biogenesis and fission, which triggers podocyte inflammation.	(118)
	*In vitro*: HPC	Lnc458/SNRPA/MYC/DDIT4	Increase mitochondrial ROS and decrease mitochondrial membrane potential	Lnc458 promotes SNRPA1-mediated MYC transactivation to upregulate DDIT4, driving mitochondrial dysfunction and podocyte apoptosis.	(120)
	*In vivo*: db/db mice *In vitro*: NRK-­52E cells	miR-124-3p/FOXQ1/Sirt4	Mitochondrial dynamics	MiR-124-3p downregulation induces FOXQ1 to inhibit Sirt4, driving mitochondrial dysfunction and DKD progression.	(122)

MicroRNAs are endogenous, non−coding, single−stranded small RNAs of approximately 22 nucleotides (nt) in length and are widely distributed among eukaryotes (114). The miR−29 family is highly conserved throughout mammalian evolution and consists of three mature members—miR−29a, miR−29b, and miR−29c—which serve as key regulators in various biological processes and diseases (115). It has been demonstrated that miR−29b is upregulated in early−stage glomeruli of DKD. Subsequent experimental evidence indicates that miR−29b downregulates PGC−1α, leading to mitochondrial dysfunction and podocyte injury, thereby exacerbating DKD progression. Thus, targeting miR−29b could serve as a potential diagnostic biomarker for early−stage DKD (116). Long non-coding RNAs (lncRNAs) are ncRNAs longer than 200 nucleotides, subject to epigenetic regulation through DNA methylation and histone modifications (117). The lncRNA plasmacytoma variant translocation 1 (PVT1), located on chromosome 8q24.21, has been associated with DKD. Zhimei et al. demonstrated that PVT1 regulates AMPKα ubiquitination via post−transcriptional interaction with TRIM56, resulting in abnormal mitochondrial biogenesis and fission, which subsequently induces podocyte inflammation and accelerates DKD progression (118). Small nuclear ribonucleoprotein polypeptide A’ (SNRPA1) is a component of the spliceosome involved in RNA processing and splicing (119). Recent studies have reported that the long ncRNA ENST00000458139 (lnc458) is significantly elevated in DKD patients and in high−glucose−induced podocytes, an effect associated with its interaction with SNRPA1. Specifically, lnc458 promotes DNA damage−inducible transcript 4 (DDIT4) expression through SNRPA1−mediated MYC transactivation, leading to mitochondrial dysfunction and podocyte injury, which collectively accelerate DKD progression (120). FOXQ1, a transcription factor within the Fox family, is characterized by a highly conserved forkhead DNA−binding domain and is known to promote epithelial−mesenchymal transition (EMT) and cancer metastasis (121). Luquan et al. identified a critical role for miR−124−3p in regulating renal mitochondrial function. Downregulation of miR−124−3p induces FOXQ1 expression in renal tubular epithelial cells, which in turn inhibits Sirt4, resulting in mitochondrial dysfunction and promotion of DKD progression (122).

Collectively, epigenetic modifications and PTMs collaboratively regulate MQC in DKD. Epigenetic regulation primarily controls the transcription and post-transcription of mitochondrial genes, whereas PTMs directly modify mitochondrial proteins. These layers interact closely. For example, ncRNAs may regulate enzymes involved in PTMs, and PTMs can influence transcription factor activity, creating a coordinated network that drives mitochondrial injury in DKD. A comprehensive understanding of these integrative networks, ideally supported by systems biology approaches and advanced sequencing technologies, will be essential for identifying more effective therapeutic targets in DKD.

## 5 Modulation of epigenetic and post-translational modifications regulating MQC for DKD therapy

We have identified a diverse therapeutic strategy targeting epigenetic and PTMs combined with MQC for treating DKD. This approach encompasses natural compounds, cell-derived therapies, modern pharmaceutical treatments, mitochondrial antioxidants, and other modalities. These interventions collectively maintain mitochondrial homeostasis, thereby improving renal function and slowing disease progression (**Table 3**).

**Table 3:** Modulation of epigenetic and PTMs regulating MQC for DKD therapy.

Therapy methods	Medication	Modification	Mechanism of action	Effect	Ref
Natural compounds	Astragalin (AG)	Ubiquitination	Drp1	AG binds Drp1 to promote its ubiquitin-proteasome degradation, inhibiting excessive mitochondrial fission and alleviating renal fibrosis.	(125)
	Isoorientin (ISO)	Phosphorylation	PI3K-AKT-TSC2-mTOR	ISO inhibits the PI3K/AKT/TSC2/mTOR pathway to reverse hyperglycemia-induced TSC2 S939 overphosphorylation, stimulating mitophagy and improving DKD.	(127)
	Echinochrome A (EchA)	Phosphorylation	PKCι/p38 MAPK AMPKα/NRF2/HO-1	EchA activates AMPK phosphorylation, preventing DKD by inhibiting PKCι/p38 MAPK and upregulating the AMPKα/NRF2/HO-1 signaling pathway.	(128)
Cell-Derived Therapies	RGD‐Treg‐Exos	ncRNA	miR‐218‐5p/TNC/TLR4/SRC/FUNDC1	Treg-Exos activate the miR-218-5p/TNC/TLR4/SRC/FUNDC1 axis to restore mitophagy and alleviate podocyte injury in DKD.	(56)
Modern Pharmaceutical Therapy	Melatonin	Phosphorylation	AMPK-PINK1	Melatonin activates AMPK to drive mitochondrial PINK1/Parkin recruitment, thereby enhancing mitophagy and mitigating renal injury.	(130)
Mitochondrial Antioxidant	Mito-tempo (MT)	Phosphorylation	PKR/eIF2α	MT reduces mtROS by inhibiting mt-dsRNA release and PKR/eIF2α phosphorylation, thereby preventing tubular injury in DN.	(132)
Chinese patent medicines	Jinlida granules (JLD)	Phosphorylation	AMPK/PGC-1α	JLD activates the AMPK/PGC-1α axis to improve mitochondrial dysfunction and podocyte apoptosis, thereby alleviating DKD.	(134)

### 5.1 Natural compounds

Astragalin (AG) is a natural flavonoid that can be extracted from various well-known edible plants, such as green tea, mulberry, and dodder seeds (123). Research indicates that AG effectively improves renal pathological damage and fibrosis in DKD mice by promoting MQC through the AMPK/PGC1α pathway, thereby exerting protective effects on the kidneys (124). Recent studies suggest that AG may inhibit excessive mitochondrial fission by promoting ubiquitin-dependent Drp1 degradation, positioning it as a potential therapeutic agent for diabetic renal fibrosis (125). Isoorientin (ISO), a natural C−glucosylated flavonoid, has garnered significant interest owing to its diverse pharmacological activities (126). Zili et al. demonstrated that ISO counteracts hyperglycemia−induced excessive phosphorylation of TSC2 at S939 and stimulates mitophagy by inhibiting the PI3K−AKT−TSC2−mTOR pathway, thereby mitigating the progression of DKD (127). Additionally, echinoderm Pigment A (EchA) is a natural bioactive compound derived from sea urchins. Studies have demonstrated that EchA improves renal mitochondrial function in DKD and enhances ATP production via activation of AMPK phosphorylation and the NRF2/HO−1 pathway. Moreover, EchA inhibits the protein kinase C−iota (PKCι)/p38 mitogen−activated protein kinase (p38MAPK) signaling axis in DKD and simultaneously downregulates the phosphorylation of p53 and c−Jun, thereby alleviating DKD pathology (128). However, current evidence is limited to preclinical studies, with important gaps in bioavailability, target specificity, and human safety data.

Future priority research should focus on dose-response studies, target validation using genetic models, and early-phase clinical trials to determine the true therapeutic potential and address the translational gaps identified in previous studies.

### 5.2 Cell-derived therapies

Over the past few decades, mesenchymal stem cells have consistently been a major focus of research. Recent studies have revealed that regulatory T cells (Tregs) can reduce podocyte injury in DKD through exosomes. Specifically, Treg−derived exosomes (Treg−Exos) were engineered to display RGD peptides on their membranes. These RGD−modified Treg−Exos bind to integrins on podocyte surfaces, delivering miR−218−5p to enhance podocyte mitophagy, reduce apoptosis, and mitigate podocyte injury by inhibiting the TNC/TLR4/SRC/FUNDC1 pathway (56). This engineered exosome approach represents an innovative cell-derived strategy; however, its efficacy and safety have yet to be tested in clinical settings, and challenges including manufacturing scalability, delivery specificity, and immunogenicity need careful evaluation.

### 5.3 Modern pharmaceutical therapy

Melatonin, a neurohormone secreted by the pineal gland, has been shown to upregulate miR−4516, which subsequently inhibits the overexpression of SIAH3 in the renal cortex during CKD. SIAH3, an E3 ubiquitin ligase, facilitates PINK1/Parkin−dependent mitophagy, thereby clearing dysfunctional mitochondria and improving renal function (129). Similarly, melatonin facilitates AMPK phosphorylation in DKD, accelerates the translocation of PINK1 and Parkin to mitochondria, and consequently activates mitophagy. This mechanism ultimately alleviates oxidative stress and reduces renal fibrosis in DKD mice (130). While preclinical data are encouraging, clinical trials specifically assessing melatonin for DKD progression are limited, and factors such as optimal dosage, bioavailability in diabetic patients, and potential hormonal side effects warrant further study.

### 5.4 Mitochondrial antioxidant

Mito−TEMPO (MT) is a widely studied mitochondrial− targeted antioxidant and a structural analog of mitochondrial superoxide dismutase (SOD). It is composed of the piperidine nitroxide (2,2,6,6−tetramethylpiperidine−1−oxyl) linked to a lipophilic triphenylphosphonium cation (TPP), which enables its membrane−potential−dependent accumulation within the mitochondrial matrix (131). Research has demonstrated that MT reduces mitochondrial double−stranded RNA (mt−dsRNA) expression and suppresses excessive mtROS production in tubular cells of diabetic mice. Furthermore, MT decreases the phosphorylation levels of PKR/eIF2α and attenuates apoptosis, thereby protecting against tubular injury in DKD (132). These benefits are confined to preclinical models; clinical applicability remains unproven, with concerns over long-term safety, tissue specificity, and effective dosing in humans.

### 5.5 Chinese patent medicines

Chinese patent medicines, a component of Chinese herbal medicine, provide advantages such as convenient use, established efficacy, lower dosage requirements, and fewer side effects compared to many Western pharmaceuticals (133). Jinlida granules (JLD) are a traditional Chinese medicine formula comprising 17 herbal ingredients. Research demonstrates that JLD effectively improves renal function, mitigates podocyte injury, and inhibits Drp1−mediated mitochondrial fission and apoptosis in db/db mice. Mechanistically, JLD enhances AMPK phosphorylation, thereby upregulating PGC−1α expression and ultimately alleviating podocyte apoptosis and mitochondrial dysfunction in DKD (134).

In summary, several strategies targeting epigenetic and post-translational modifications to regulate mitochondrial quality control (MQC) have demonstrated promising effects in preclinical DKD models, including natural compounds (e.g., astragalin, isoorientin), cell-derived exosomes, melatonin, Mito-TEMPO, and Jinlida granules. Recently, Du et al. reported a novel red blood cell membrane-encapsulated mitochondrial transplantation approach that effectively rescued mitochondrial dysfunction in models of mitochondrial disorders and Parkinson’s disease (135). Although this strategy has not been tested in DKD, it offers a promising complementary approach for MQC restoration. Nevertheless, most current interventions remain preclinical, with key limitations in target specificity, bioavailability, dosage optimization, and clinical translation.

## 6 Conclusions and future perspectives

Kidney disease represents a major global health challenge, affecting approximately 10% of the world’s population (136). Among these, DKD has emerged as a major global public health issue, with its prevalence increasing year by year. This condition significantly elevates patients’ risk of cardiovascular events, exacerbates the burden on healthcare systems, and leads to higher mortality rates (137, 138). As a key metabolic organ, the kidney requires substantial energy expenditure to actively maintain systemic metabolic balance, plasma hemodynamic stability, electrolyte and water homeostasis, nutrient reabsorption, and hormone secretion (139). The majority of renal ATP is generated through mitochondrial oxidative phosphorylation, with only a minor fraction derived from glycolysis (140). Mitochondrial dysfunction is closely linked to the initiation and progression of DKD—an association that has long been recognized. This review first systematically summarizes the core regulatory mechanisms of MQC in DKD, covering key processes such as mitochondrial biogenesis, dynamics, and mitophagy. Second, accumulating evidence in recent years indicates that epigenetic modifications and PTMs not only play significant roles in DKD but also finely tune MQC. At different stages of MQC, one or more epigenetic and PTM mechanisms often act in concert to drive disease progression. Therefore, this article emphasizes the crucial role of epigenetic modifications and PTMs in regulating MQC during the pathogenesis of DKD and elucidates their interactive regulatory relationships within this context. Finally, we summarize related therapeutic strategies based on this regulatory network, aiming to provide new insights and potential directions for the prevention and treatment of DKD.

In recent years, despite significant advancements in DKD research, many unresolved questions remain regarding its pathological mechanisms. The regulation of MQC by epigenetic modifications represents an emerging field that is still in its early stages. However, current research in this area remains largely focused on classic modifications including methylation, ubiquitination, phosphorylation, glycosylation, and acetylation, while attention to newer modifications such as propionylation, butyrylation, and succinylation is relatively limited. Furthermore, epigenetic regulation is inherently complex. For instance, acute kidney injury (AKI) is not only associated with mitochondrial dysfunction but also involves processes such as acetylation and ferroptosis (141). Most existing studies tend to focus on single mechanisms or pathways, and a systematic, integrative analysis of how multiple epigenetic and post-translational modification networks coordinately regulate MQC in DKD is still lacking. Looking ahead, with the rapid development of sequencing technologies, single-cell omics, and computational biology, as well as the growing availability of public databases and large biobanks, research in this field is poised for substantial expansion in both depth and breadth. For example, studies have already utilized DNA methylation markers to reflect the progression of DKD and renal functional status (142). Integrating single-cell epigenetic technologies may enable more precise identification of disease-specific biomarkers at early stages, thereby facilitating early diagnosis and intervention. Additionally, mitochondrial base editors (mitoBEs) such as DdCBEs and TALEDs have shown promise in correcting mtDNA mutations in non-renal diseases (143, 144). These tools offer precise editing without double-strand breaks and may help address mitochondrial defects in DKD, though their delivery, off-target effects, and renal safety require further investigation. It is noteworthy that most current findings remain at the basic research stage. Translating these molecular insights into effective clinical diagnostic and therapeutic strategies represents a key challenge for future work.

In summary, although epigenetic and post-translational modifications appear to play important regulatory roles in MQC during DKD pathogenesis, most current evidence remains associative and is largely derived from cell and animal models. Caution is therefore warranted when extrapolating these findings to human DKD. Further rigorous studies, particularly in human systems, are needed to establish their causal roles and therapeutic potential. A deeper understanding of their fine-tuned control over MQC may ultimately provide a foundation for novel preventive and therapeutic strategies.
